# Process research: compare and contrast the recovery-orientated strengths model of case management and usual community mental health care

**DOI:** 10.1186/s12888-021-03523-5

**Published:** 2021-10-28

**Authors:** Samson Tse, Catalina S. M. Ng, Winnie W. Y. Yuen, Iris W. K. Lo, Sadaaki Fukui, Richard J. Goscha, Eppie Wan, Stephen Wong, Sau-Kam Chan

**Affiliations:** 1 Department of Social Work and Social Administration, The University of Hong Kong, Pok Fu Lam, Hong Kong; 2 Department of Early Childhood Education, Education University of Hong Kong, Tai Po, Hong Kong; 3 Department of Counselling and Psychology, Hong Kong Shue Yan University, North Point, Hong Kong; 4 School of Social Work, Indiana University – Purdue University Indianapolis, Indianapolis, Indiana, USA; 5 California Institute for Behavioral Health Solutions, Center for Mental Health Research and Innovation, California, USA; 6 Lok Hong Integrated Community Centre for Mental Wellness Wong Chuk Hang Complex, Tung Wah Group of Hospitals, Wong Chuk Hang, Hong Kong; 7 Caritas Wellness Link – Tsuen Wan, Integrated Community Centre for Mental Wellness, Caritas Hong Kong, Tsuen Wan, Hong Kong; 8 Integrated Community Centre for Mental Wellness (Eastern), Baptist Oi Kwan Social Service, Shau Kei Wan, Hong Kong

**Keywords:** Strengths intervention, Process evaluation, Mental health, Recovery

## Abstract

**Background:**

The strengths model of case management (SMCM), which was developed by Rapp and Goscha through collaborative efforts at the University of Kansas, assists individuals with mental illness in their recovery by mobilizing individual and environmental resources. Increasing evidence has shown that the utilization of the SMCM improves outcomes, including increased employment/educational attainment, reduced hospitalization rates, higher self-efficacy, and hope. However, little is known about the processes through which the SMCM improves outcomes for mental health service users. This study explores the views of case workers and service users on their experience of providing or receiving the SMCM intervention.

**Methods:**

A qualitative design was employed using individual interviews with service users and case workers drawn from two study conditions: the SMCM group and the control group (treatment as usual). For both study conditions, service users were recruited by either centres-in-charge or case workers from integrated community centres for mental wellness (ICCMWs) operated by three non-governmental organizations (NGOs) in different districts of Hong Kong. Through purposeful sampling, 24 service users and 14 case workers from the SMCM and control groups joined the study. We used an inductive approach to analyse the qualitative data.

**Results:**

We identified two overarching themes: service users’ and case workers’ (1) perceptions of the impacts of the interventions (SMCM and control group) and (2) experiences of the interventions, such as features of the interventions and the factors that facilitated the outcomes. The results showed that there were improvements in the functional recovery of the SMCM group in areas such as employment and family relationships, how self-identified goals were achieved, and how service users gained a better understanding of their own strengths and weaknesses. Regarding their experience of the interventions, participants in both the SMCM group and the control group reported that a good relationship between service users and case workers was vital. However, some concerns were raised about the use of SMCM tools, including the strengths assessment and the personal recovery plan (PRP) and the risk of case workers being subjective in the presentation of cases in group supervision sessions.

**Conclusion:**

The results were promising in terms of supporting the use of the SMCM, with some refinements, in mental health services for Chinese clients.

**Trial registration:**

The Australian New Zealand Clinical Trials Registry (ANZCTR), ACTRN12617001435370, registered on 10/10/2017.

## Background

The strengths model of case management (SMCM), which was developed by Rapp and Goscha [1, 2] and faculty members and students at the School of Social Welfare of the University of Kansas in early 1980s, is designed to be an alternative to the pathology and problem oriented approaches in psychiatric rehabilitation. The SMCM is based on six major principles [2]: (1) the focus is not on service users’ deficits but their strengths; (2) service users can learn, grow and change; (3) the community is perceived as an oasis of resources; (4) service users direct the helping process; (5) the worker–service user relationship is vital; and (6) the prime setting of the interventions is the community. Case management refers to the process of identifying needs, designing a service plan and monitoring progress to bring about positive outcomes [3, 4]. The SMCM is distinct from traditional mental health approaches that put the emphasis on problems, pathology and diagnosis, hold low expectations of the achievements of mental health patients in their life, and often use stabilization as one of the key measurements of success [2]. At the individual level, the SMCM stresses the importance of personal goals, encourages positive risk taking and values the goals as an important part of the recovery and is guided by clear fidelity standards. At the systems level, the SMCM (1) emphasizes case workers and group supervisions which come up with solutions for service users and (2) involves service users and their families and staff at all levels. Tse, Davis, and Li [5] stressed that compared with other models of case management in the field of mental health, the strengths model is relatively well defined in different areas, such as assessment, data collection, therapeutic process, quality assurance, and evaluation. Vanderplasschen and colleagues [6] compared three commonly used models (the brokerage/generalist model, assertive community treatment/intensive case management, and the clinical/rehabilitation model) with the SMCM for substance users, and they concluded that these case management models can be distinguished on the basis of the levels of service provision, client participation, and case worker management. Specifically, the distinctive characteristics of the SMCM lie in the strengths and empowerment approach, which differs from assertive community treatment/intensive case management, which uses the comprehensive approach. Only the SMCM and the clinical/rehabilitation model offer both coordination and service provision, while the brokerage/generalist model offers mainly coordination. Regarding group supervision, only the SMCM requires case workers to follow specific steps and focus on the development of resources and goals for service users. The characteristics of the different models of case management are summarized in Table 1. Another distinctive feature that makes the SMCM unique is the use of strengths tools to structure/orient recovery-based practices; most of the existing case management models are idiosyncratic and, with the exception of the assertive community treatment model, may not have any specifically defined structures or tools.

**Table 1:** Main characteristics of distinguished models of case management (adapted from [6], p. 95)

Models				
Characteristics	Brokerage and Generalist Case Management	Assertive Community Treatment and Intensive Case Management	Strengths-based Case Management	Clinical Case Management
Distinctive characteristic	Coordination	Comprehensive approach	Focusing on strengths and empowerment approach	Case worker as role-model and therapist
Outreaching, service provision at home	Not the priority	Yes	Yes	Yes
Coordination or service provision	Mainly coordination	Service provision	Coordination and service provision	Coordination and service provision
Case worker’s or multidisciplinary team’s responsibility	Case worker	Team	Case worker	Case worker
Growth or stabilization of clients	Mainly stabilization	Stabilization and growth	Stabilization and growth	Mainly stabilization
Group supervision	No specific information on how it is conducted	No specific information	Following specific steps and focusing on resources development and goals surrounding the client	No specific information
Average caseload	35+	15+	15+	10+

When using the SMCM with particular reference to service users with mental health problems, the service users establish their goals for their life (goal determination) and are guided to recognize their strengths (e.g., talents, resources, etc) to achieve these goals (strengths assessment). Regarding resources, the environment or community in which service users live can be rich in resources that can help them reach their goals (resources from the environment/community). Based on strengths-based approaches, the SMCM seeks to increase service users’ level of hope as hope can be realized by finding their own strengths and through empowering relationships with others, and with the community and culture [7]. The SMCM allows service users to improve their perceptions of their abilities and increase their confidence and enhances their opportunities to make and act on their own meaningful choices [8]. Thus, the self-empowerment and self-determination of service users are well supported so that they can have control over their well-being. The existing literature (including the SMCM-specific studies) supports the effectiveness of generic strengths-based case management in improving client outcomes, such as reducing hospitalization, improving physical and mental health, increasing employment, and increasing social support and satisfaction with life [1, 9–17]. Aside from client outcomes, Tsoi and colleagues [18] found that the SMCM was effective for reducing emotional exhaustion among case workers. However, very little is known about how individuals receiving an SMCM intervention perceive their experience [19]. What is also missing from the literature is the processes through which the SMCM improves outcomes for mental health service users. Process research helps delineate any factors that may affect the implementation and maintenance of an intervention [20–23]. The results of process research can help researchers improve our understanding about the outcomes of an intervention and inform future refinements of the intervention [24]. Therefore, through qualitative interviews, we aim to explore service users’ and workers’ views on their experience of receiving or providing interventions (either SMCM or control group).

### A review of SMCM studies using the qualitative method

We conducted a review of the qualitative studies on the SMCM. The academic articles were identified by searching the electronic databases PubMed, Web of Science and EBSCOhost, including ERIC, MEDLINE and PsycINFO, which cover the period 2000 to 2020. Table 2 summarizes the studies that have investigated the SMCM using qualitative research. The results showed that only six studies have been conducted: five studies using Western samples and one study by Tse, Divis, and Li [5] using Chinese participants. Tse and colleagues [5] examined the perspectives of Chinese service users in New Zealand on their experience of the use of the SMCM model in facilitating their mental health recovery. However, as these Chinese service users had been residing in New Zealand for different lengths of time, it is difficult to conclude the extent to which Western culture had impacted them. In addition, how individuals conceptualize strengths is culturally bound because the conceptualization of strengths is ‘culturally defined through linguistics, metaphors, icons and folklore traditions’ ([29], p.3). Chinese people perceive their strengths as ever changing, universal and dialectical and shaped by their upbringing or family traditions. Moreover, Chinese people are deeply influenced by traditional cultures such as Taoism and Confucianism (the Doctrine of Mean), so they are more reserved in stating their strengths and successes. Therefore, it is not surprising that the results from the West might be different from studies conducted in a Chinese (or, more broadly, Asian) context.

**Table 2:** Summary of using qualitative studies on strengths model case management (SMCM)

Author(s) (Year)	Country	Sample size	Aims	Findings
Schuetz et al. (2021) [25]	U.S.A.	34 participants (28 case managers, five supervisors and one children’s service director)	• Understood the process of implementation • Explored how the adapted strengths model for case management impacted the workers and their work with young people	• The model impacted on organizational process and culture, the provision of services and adaptations of the model for young people • Participants expressed that they were satisfied with the model
Schuetz et al. (2019) [26]	U.S.A.	34 participants (28 case managers, five supervisors and one children’s service director)	• Explored how SMCM impacted the workers’ work with young people and youth outcomes	• Three themes were: model design and delivery, intermediate impact and long-term outcomes • There was overall satisfaction with the model
Petrakis et al. (2013) [27]*	Australia	• The number of participants was not mentioned • Three sites (the intensive residential CCU and the two community CCT sites) joined	• Evaluated the implementation fidelity of group supervision in the SMCM	• There was a high fidelity for group supervision for group interaction, client work and by case managers • A standardized approach to group supervision process and documentation facilitated fidelity in implementation
Tse et al. (2010) [5]	New Zealand	35 participants	• Examined how SMCM was perceived from the Chinese cultural perspective • Identified the barriers reported by practitioners when they applied the SMCM	• The focus on personal and collective strengths and pragmatic approach were regarded by participants as distinctive features of the model • The service user participants regarded the strengths model as helpful in assisting their settlement and integration into society • Practitioners faced with three challenges: passive role played by service users, difficulties in understanding the concept of strengths and service users with complex needs
Redko et al. (2007) [28]	U.S.A.	26 substance abusers	• Explored how people with substance abuse perceived the working alliance with case managers	• A positive working alliance was important to build trust, self-worth and self-esteem • The personal qualities of the case manager and the nature of the client-case manager relationship were crucial • Two principles of SMCM: personal control over goal setting and an emphasis on strengths
Brun & Rapp (2001) [19]	U.S.A.	• Two project case managers • 10 individuals were experts who joined the Case Management Enhancements Project (CME)	• Explored the participants’ perceptions of SMCM • Compared the participants’ perceptions with the key principles of SMCM	• Individuals’ responses to the SMCM (acceptance of strengths, initial mistrust of the strengths-based approach and hold on to strengths and deficits at the same time) • Individuals’ responses to the professional relationship (acceptance of the relationship, do not need the relationship and felt guilty when failed)

Note: * a mixed method was used

Apart from the cultural difference, the structural compatibility (e.g. caseload size and ratio of supervisor to case worker) is also different between service contexts. For instance, the structure of mental health services in Hong Kong is different to that in the USA. The caseload in mental health services in Hong Kong is relatively high according to a report released by the Hospital Authority in Hong Kong in 2016, which stated that the ratio of case workers to service users was around 1 to 47.

Besides, since most SMCM studies have been conducted in the Western context, the use and outcomes of the SMCM lack cultural sensitivity to non-Western cultures. Therefore, we made preliminary cultural adaptations, such as translating the forms used in the strengths assessment and personal recovery plan (PRP) and using local terms and examples to explain the concept of strength, before we implemented the randomized controlled trial (RCT). The process evaluation can provide us with insights into the effectiveness of such cultural adaptations. Hence, filling the gap in our understanding of the effects and experiences of the SMCM is the goal of the present study.

## Method

### Study design and setting

The present study was part of a larger RCT study that aimed to evaluate the effectiveness of the SMCM compared with usual treatment. Individual interviews were conducted with service users and case workers in two study conditions: the SMCM group and the control group (treatment as usual). The study received ethical approval from the Human Research Ethics Committee of The University of Hong Kong (HRECNCF: EA1703078). For both study conditions, service users were invited to participate by either the centres-in-charge or case workers from ICCMWs operated by three NGOs in different districts of Hong Kong. Each centre made both service groups available to service users who joined the centre voluntarily.

#### SMCM group

The SMCM group used five indispensable tools: strengths assessment, PRP, group supervision, field monitoring and fidelity review. The strengths assessment identified the strengths of service users, niches in the community and other attributes, in particular, self-efficacy, hope and the resources provided by the family and community. The PRP utilized the information from the strengths assessment to devise a plan that included recovery goals. The steps for constructing recovery goals should be small, specific and measurable and should be co-planned by service users and case workers. The aim of the group supervision was to provide regular structured supervision for case workers to assist service users in achieving their goals. Case workers were provided with field mentoring to improve their practical SMCM skills and approach towards service users. The SMCM fidelity review was conducted every six months to monitor whether the high-fidelity activities took place as expected. The SMCM group achieved an overall fidelity score of 36 (out of 45) on the fidelity scale (including an average rating of four out of five in each of the three core areas: structure, supervision/supervisor and clinical/service), which was considered as achieving high fidelity. The fidelity review data were collected through interviews with staff and service users, site observations, and reviews of the SMCM tools and charts about the service users.

In addition to receiving their usual services (see section below on the control group), the service user participants received individual SMCM sessions lasting about 30 min every fortnight, guided by the strengths assessment and PRP. Case workers helped the service users to identify recovery goals that were meaningful to them. Case workers and their supervisors had previously received two days of SMCM training provided by members of the University of Kansas School of Social Welfare. Workers were supported by weekly strengths-based supervision sessions at the ICCMWs, and supervisors attended ongoing monthly group supervision led by RG (SMCM’s founder) via Zoom.

#### Control group

The service user participants in the control group received their centre’s usual services (treatment as usual), which included recovery-based interventions (e.g., wellness management, peer-support services), leisure/hobby groups, medical appointments and general community activities (e.g., outings, social gatherings). Case workers had fortnightly contact with service users in person or on the phone, and this contact included tangible work, such as following up on matters related to welfare benefit and accommodation or sending greeting messages to check on the service user’s condition. Case workers did not apply any SMCM tools (e.g., strengths assessment, PRP). Case workers in both groups received either individual or group supervision (one or two sessions per month). Table 3 shows the number of case workers and service users in each group, and Table 4 summarizes the differences between the SMCM group and the control group.

**Table 3:** Qualitative interviews involving case workers and service users in SMCM group and control group

		SMCM group	Control group
ICCMW-centre A	Case workers	3	2
	Service users	6	6
ICCMW-centre B	Case workers	2	2
	Service users	3	3
ICCMW-centre C	Case workers	3	2
	Service users	3	3
Total		20	18
Total no. of case workers	14		
Total no. of service users	24

**Table 4:** Key characteristics of SMCM intervention and control groups

Dimensions	SMCM Group	Control Group
Participants in both groups will attend psychiatric outpatient appointment (if any) and regular programs in the Community Mental Health Centre e.g., community meeting, exercise class		
**Intervention Integrity & Infrastructure**	Ensures a supportive strengths model context through the *Fidelity Scale*, which was designed to assess the adequacy of SMCM implementation in three core areas: structure, supervision/supervisor, and clinical/service.	No routine fidelity assessment for the implementation of recovery-oriented services.
**1. Structure**	Has specifications about caseload ratios and percentage of community contacts.	No specific requirement.
**2. Strengths-based Supervision**	Field mentoring and group supervision: provide support and affirmation, ideas and learning. Group supervision following specific steps: ✓ The presenting staff hand out service users’ strengths assessments and specify the help needed from the group. ✓ The team are to clarify the assessment and brainstorm ideas. ✓ The presenting staff review the ideas and state the next steps.	Adopt the existing supervision arrangements.
**3. Clinical/ Service** **a. Strengths Assessments**	Collects information on personal and environmental strengths using the *Strength Assessment* tool as the basis of work. Assessment is an ongoing process. Domains in daily living, assets, employment/education, supportive relations, wellness/health, leisure, spirituality/ culture.	No specific tool for conducting initial assessments. Unclear how it will focus on assessing people’s strengths.
**b. Personal Recovery Plans**	Creates a mutual agenda for work, focusing on achieving the goals that the person has set. Writes down the person’s goals (passion statement) and plan specific steps (short-term goals) to achieve the goals in the *Personal Recovery Plan*.	Work on specific goals. No specific tool.

### Participants

The participants were persons attending the ICCMWs who had been diagnosed by a psychiatrist as having either suspected mental health problems or mental illnesses, including major depressive disorder, anxiety disorder, bipolar disorder and psychotic disorder. The inclusion criteria for participation were as follows: aged 18 or above, ethnically Chinese, Cantonese speaking, and able to give written informed consent. The exclusion criteria applied to those service users identified by case workers as being likely to engage in immediate risk behaviour, such as suicide and/or violence, and/or affected by overt symptoms and unable to sustain a meaningful conversation for more than 10 min. The case worker participants were case workers of individuals in the SMCM or control groups at the ICCMWs. After explaining the purpose of the study and their rights, written consent was obtained from the participants. Incentives in the form of supermarket coupons (worth HK$50 or US$6.5) were given to the service user participants for the individual interview. The case worker participants did not receive incentives.

**Fig. 1:**
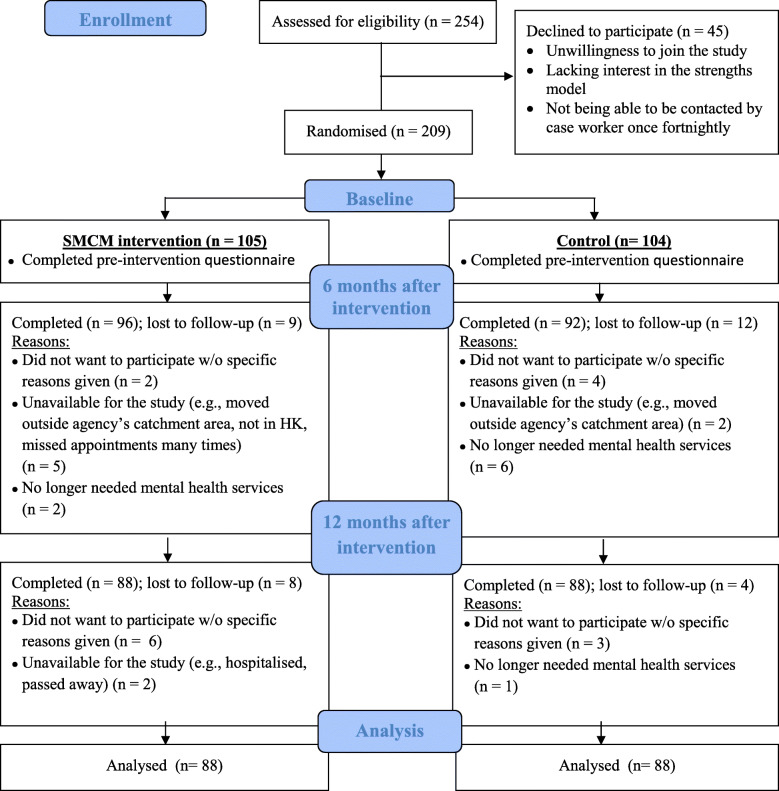
CONSORT diagram reflecting the flow of service user participants through the original randomized controlled study

Through purposeful sampling, a total of 24 service users and 14 case workers (two community-based nurses, eight social workers and four welfare workers) from both groups were invited to participate in this study (Please see the CONSORT flow diagram, Fig. 1). A total of 18 females (75%) and six males (25%) participated in the study. They ranged in age from 26 to 67 years, with an average age of 49.4 years (SD = 11.2) and a median age of 54.0 years. Their recorded diagnoses in the case files included schizophrenia (*n* = 12; 50%), depression (*n* = 9; 37.5%), bipolar disorder (*n* = 2; 8.3%) and adjustment disorder (n = 1; 4.2%). Regarding time since the first onset of their condition, the range was between one year and 32 years, with an average of 11.5 years (SD = 9.3). In terms of the number of years of experience working with individuals with mental illness, the case worker participants in the SMCM group had between 0.5 and 6 years (mean years = 4.3; SD = 1.9) of experience and those in the control group had between 0.5 to 20 years (mean years = 8.4; SD = 7.6) of experience. An independent-samples t-test was conducted to examine age and time since onset of illness for the SMCM and the control group. There was no significant difference between the SMCM group and the control group in terms of age (SMCM group: M = 52.0, SD = 9.3; control group: M = 46.8, SD = 12.7; t (22) = 1.15, *p* = .26) and onset of illness (SMCM group: M = 11.50, SD = 10.08; control group: M = 11.42, SD = 9.42; t (22) = .021, *p* = .98). The results also showed no significant difference between the case workers of both groups in terms of years of working experience (SMCM group: M = 4.29 (SD = 2.08) vs control group: 8.43 (SD = 8.24); t (6.76) = − 1.29, *p* = .24).

### Data collection and management

Each interview was conducted by the same person (IWKL), lasted between 30 and 90 min, and was carried out in an interview room at the ICCMWs. The data collection was directed by a semi-structured interview guideline which included the following domains: perceptions of the benefits resulting from the interventions and any challenges in providing or receiving the services. The interview questions for service users included the following: Can you share with us the impact(s) (desirable and less desirable) that the SMCM has had on your own personal growth or your personal recovery journey? How did the impact happen? The interview questions for the case workers included the following: What have been your experiences in using the SMCM? Any differences compared with your previous working experience?

Prior to conducting the interviews, consent was obtained from all participants for the interviews to be audio-recorded. The audio recordings were saved in an encrypted folder and were transcribed verbatim into Chinese by professional transcribers for subsequent analysis and interpretation. All identifiable personal information was removed from the text to ensure confidentiality. IWKL performed quality checks by comparing the recordings to the transcripts. Only the direct quotes cited in the present manuscript were translated into English. In addition to the audio recording, notes were taken during each interview in case there were any technical problems with the recording. Data collection was discontinued when no new codes occurred in the data or data saturation was achieved.

### Data analysis and research rigor

An inductive approach, as proposed by Thomas [30], was used in the qualitative analysis for its simplicity in linking the data analysis to the specific research questions. The transcripts were first studied closely line by line, with notes written on the transcripts. Then, upper-level categories were created and coded by the researchers. The categories were compared and contrasted to search for consistencies, contradictions, and interrelationships. For each category, direct quotes from the participants were also used to represent the emerging themes.

In order to confirm the reliability and validity of the coding process, two researchers (ST and IWKL) read all the interview transcripts independently to familiarize themselves with the data and then met several times to discuss and code the interviews using the semi-structured interview guideline as a basic framework. They compared their initial coding of two identical scripts and refined the analysis framework (e.g., what is special about the service experiences, anything about the SMCM or the control group and factors that might affect the outcomes). ST and IWKL met again to compare the coding scheme per the refined analysis framework after reviewing three more identical scripts independently prior to the meeting. Then, they confirmed the analysis framework, where the inter-rater reliability showed an 88.2% agreement level (15 out of 17 codings completed). After ST coded the remaining interviews using the agreed coding system, ST and IWKL reviewed and refined the results, producing two overarching themes, namely (1) perceived impacts of the services received and (2) service users’ and workers’ experience of the mental health interventions (SMCM or usual care). The sub-themes were determined on the basis of two main considerations: (1) the similarities and differences between the SMCM and control groups when the same interview topics (e.g., who may benefit from the intervention) were presented to the research participants during the interviews; (2) the saliency and richness of the material that emerged from the data. One typical example noted by the researchers was that compared with the service user participants from the control group, the service user participants from the SMCM group tended to provide relatively more vivid and thick descriptions about the impacts of services on their goal achievements. Closely related to the “salience” consideration was the counting of the emergent material. For example, compared with the workers in the control group, the workers in the SMCM group used a greater number of adjectives or illustrative phrases in describing their interventions (e.g., “*using the strengths assessment*”, “*being present with the client*”). To enhance the rigor of the research and to ensure the validity and credibility of the analytic interpretations, a presentation about the results was delivered in a sharing session involving the staff of the three ICCMWs. Their discussion and comments further refined the analysis and enhanced the accuracy of the results.

## Results

The results fall into two main themes: (1) impacts of the community mental health interventions as perceived by service users and case workers and (2) experience of the interventions, such as features of the interventions (SMCM and control groups) and the factors associated with the outcomes. The results in terms of the themes and sub-themes are summarized in Table 5.

**Table 5:** Summary of themes and sub-themes

**1. Impacts of interventions**		
**Common impacts:** Improved motivation level, better management of one’s emotion, mental symptoms and negative thoughts		
**Different impacts:**	**SMCM group**	**Control group**
**Domain of improvement-**	Improvements in functional recovery e.g., finding paid employment, widening social circle, more contact with family members, and adopting a more planful approach to achieve one’s goals	General improvement
**Account of the impacts-**	Vivid, detailed, rich accounts of how recovery goals were set and eventually achieved	Tended to be straightforward
**One’s own strengths-**	Better adjusted to understand and accept one’s strengths, weaknesses and shortcomings	No similar comments were found
**2. Experience of the intervention**		
***2.1 Relationship between service user and case worker:*** Both SMCM and control group service users appraised the case workers as very helpful and approachable, having regular contact, caring like a family members.		
	**SMCM group**	**Control group**
**Context-**	• Helping service users gain hope and a sense of satisfaction by supporting them to achieve their identified goals, showing a genuine appreciation of users’ strengths and a curiosity to explore their talents and skills	• Being person-centered in therapeutic relationship with service users, showing empathy and trust towards them
**Concerns-**	• Once-a-fortnight contact between case worker and service user was too much for user and the potential to build dependence on case worker	• Did not mention any particular concerns
***2.2 Who would benefit from the interventions?*** Service users’ characteristics were important in determining whether they would benefit from the respective interventions. Both SMCM and control group service users needed to have some insight, willingness to engage in conversations about one’s own recovery process and have some social support.		
	**SMCM group**	**Control group**
**Service users need to have-**	• Stable mental state (e.g., no severe depressive or hypomanic symptoms) • Adequate communication skills	• Good adherence to medications regime • A “normal life”, meaningful daytime engagement • Life skills • Trust in the case worker • Opportunity to make own decisions
***2.3 Specific tools used in the interventions:*** Control group participants did not mention any particular features of the interventions. **SMCM workers commented about-** • Found the personal recovery plan very helpful for both case workers and service users • The service users had mixed feelings about how the workers used the strengths assessment and personal recovery plan • The importance of taking part in group supervision where workers could learn from each other and find optimal solutions to the problems they face. Meanwhile, the group discussion was influenced by the worker’s understanding and perception of the user		

### Impacts of interventions

Closer examination of the narratives regarding the impacts of the interventions provided in the SMCM and control groups revealed both similarities and differences across the two interventions. The common improvements across the two groups include improved motivation level in participating in various activities, such as physical exercise and psychoeducational class. Other benefits included having better ability to manage one’s emotions, mental symptoms and negative thoughts. However, participants from both groups reported that their problems (e.g., indecisiveness, poor concentration) were not improved by the interventions. Below is an example:

“*One of the problems that I cannot solve is work. I do not know why I give up so easily. I do not know whether this is a personality problem. In some jobs, I am eager to go back to work at first, but after a few days, I wouldn’t want to work because my mood would have changed. I don’t know why it changes so quickly, and I haven’t been able to solve this problem*.” (Service User: 190723_T027_KKF_Control_19)

There were three distinctive differences between the SMCM group and the control group in terms of the impacts of the interventions. First, compared with the control group, service user participants in the SMCM group tended to mention improvements in their functional recovery more, such as finding paid employment, widening their social circle, making more contact with family members or adopting a more planful approach to achieve one’s goals.

“ … *all along I did not like to plan, just do it when I want. I do not like planning. Why bother planning if I would fail anyway–that is too hard for me … but now I am more active in facing the problems … (on the topic of goals) yeah in the (non-SMCM-based) community centre I attended in the past, I was not asked about my ideas, what I wanted to achieve or learn, no one knew really, not even my wife.*” (Service User: 181024_T047_WYK_SMCM_59–60 & 66–67)

Second, service user participants in the SMCM group offered vivid, detailed, rich accounts of how their recovery goals were set and eventually achieved.

*“Specifically, when we were talking about certain thoughts – I have my own way of thinking – my case worker would help me to try another way of thinking. … Overall, my case worker provided me with some other options without setting limits for me. She taught me not to care too much whether any business transactions could be done, not to compare the number of people at other stalls. Instead, I should experience the process. She provided me with feedback. Finally, my case worker asked me how the activity was. I felt my experience had been broadened, and I felt like I had cleared a hurdle.”* (Service User: 190816_B050_WWH_SMCM_17–21)

Participants in the SMCM group expressed that even though not all goals had been achieved, it was important to feel hopeful.

*“It (SMCM) is helpful. That is, although it (achievement of goal) sometimes did not work, there is hope … although I can’t achieve the goal, I learn how to think differently, to no longer dwell on the failures, skewing to one side (the unpleasant experience)”* (Service User: 181024_T084_LHT_SMCM_78 & 83–84)

Third, the participants in the SMCM group not only referred to learning more about their strengths and talents, growing in confidence, and having a positive outlook about life in general but also elaborated on how they had become better adjusted to understand and accept their strengths, weaknesses and shortcomings. No similar comments were found in the control group.

*“Experience … My experience of this year was that I have accepted my own weaknesses and discovered my strengths. The biggest change is that when a mistake is made, it is no longer that I am right and others are wrong. I make mistakes; I accept myself as I am. Even though people made mistakes, I accepted them. This is the change.”* (Service User: 190813_C070_LYW_SMCM _88)

### Experience of the intervention

This section of results was based on three discussion topics: How did the service users and case workers characterize the interventions? Who could benefit from the interventions? What are the three or four factors that may be related to the intervention outcomes?

#### Relationship between service user and case worker

When case workers were asked about what stood out for them about the mental health interventions, the first feature case workers from both the SMCM group and the control group mentioned was the therapeutic relationship between the worker and the service user. The workers in the SMCM group recalled how they helped service users gain hope and a sense of satisfaction by supporting them to achieve their identified goals. The workers showed a genuine appreciation of users’ strengths and a curiosity to explore service users’ talents and skills. The workers in the control group talked about being person centred in their therapeutic relationship with users, showing empathy and trust towards them. The control group’s workers appraised and affirmed the determination of the service users.

*“For example, job hunting … She was successful in getting the job. The manager was very nice and asked her to work the next day … She worked for less than five hours … If there is an opportunity, I will still let her try. If it does not work, we can use other means.* (Case Worker: 190725_TS05_Control_132)

The findings that emerged from the SMCM group were different from those that emerged from the control group in terms of how the therapeutic relationship was related to the goal-setting and goal-achieving processes.

*“The service users also talked more about what they wanted. In addition, their decisions should be respected. For example, Mr. ABC (service user’s name) was not sure whether to look for a job or retire. He also asked whether retirement was a good decision. I did not answer whether it was a good decision or not; instead, I asked what he wanted. He said that he wanted to have good quality of life. Then, I respected his decision. It is not a must to work. So, we discussed life and having some kind of goal after retirement. In my opinion, these are the most important points.”* (Case Worker: 190809_CS03_SMCM_28)

One distinctive comment was found only in the SMCM group, where two workers felt that the once-a-fortnight contact, which is one of the fidelity review criteria, was rather demanding on service users because some of them already had busy schedules. Furthermore, such regular meetings may lead to service users developing a dependence on the SMCM workers.

*“I think the biggest obstacle is time. For some clients who are working, meeting once every two weeks is difficult. When I call them, it sounds like they are very tired. I feel something is not quite right … once every two weeks is difficult. For those clients who work, I will do it once a month.”* (Case Worker: 190712_CS21_SMCM_96)

*“However, sometimes I am not sure whether it (referring to the client’s apparent dependence on the worker) is because we have meetings too often. I feel that the clients may be dependent on us … ”* (Case Worker: 190809_CS03_SMCM_3)

The service user participants from both the SMCM group and the control group readily shared their opinions about many qualities associated with workers in the therapeutic relationship, such as caring like a family member, having regular contact, and being very helpful and approachable. The only apparent difference between the two groups was that the service user participants in the SMCM group elaborated much more when describing the worker–service user relationship, which resulted in very rich and thick narratives. The following account about how a worker helped a user find different ways to cope with work-related stress shows that a very deep relationship existed between the worker and the service user (T047/WYK_A73-80p14–16).

*“She mentioned that I had to adjust my rest time and showed me how to improve my confidence, how to use methods to sleep better. It was because I suffered from insomnia at that time. My case worker did not want me to rely on sleeping pills, so she taught me to drink a glass of hot milk or something similar to make me sleep better and how to better communicate with others. Ms ABC (worker’s name) helped me a lot. Previously, it was easy for me to lose my temper. When someone said something that was not nice, I would take exception immediately. Now I think about what I want to say, whether it will hurt others.”* (Service User: 181024_T047_WYK_SMCM_50)

#### Who would benefit from the interventions?

The worker participants in the SMCM group and the control group believed that service users’ characteristics were important in determining whether they would benefit from the respective interventions. The difference between the two groups was that compared with their counterparts in the control group, the worker participants in the SMCM group mentioned less requirements in regard to who would benefit from the interventions (see 2.2 ‘Who would benefit from the intervention?’ in Table 5).

#### Specific tools used in the interventions

The workers in the control group did not mention any particular features of the interventions, but the worker participants in the SMCM group gave extensive accounts of the interventions they provided. It was mentioned that SMCM workers need to adhere to the model’s protocol, such as using the strengths assessment and PRP. The workers are expected to support service users to set meaningful goals, provide them with options and help them to devise small steps to reach the set goals. One worker participant highlighted the importance of taking part in strengths-based group supervision where workers could learn from each other and find optimal solutions to the problems they face. While not disagreeing that group supervision was one of the SMCM’s important tools, one worker participant cautioned that the group discussion was heavily influenced by the case worker’s understanding and perception of the user. The material presented and the subsequent discussion could well be biased or focused only on certain aspects.

*“But there is a difficulty in SMCM group supervision because when we do group supervision, most of us would never have seen the client before. The case worker will present how he/she sees the client. This is an indirect way for others to familiarize themselves with the client. Everyone can interpret the client in different ways. That’s why when I need to introduce a client I am responsible for, I may have my own perception of the client, so my presentation of the client may not totally represent who the client really is. Actually, sometimes it’s a bit difficult.”* (Case Worker: 190809_CS03_SMCM_20)

The case workers found the PRP very helpful for both staff members and service users.

*“I think that the PRP helps us to reach a consensus … it is a basis for us to discuss together and decide on a plan of action. Whether we show the PRP form to a client largely depends on the client. We write down the PRP and show it to the client so they clearly know what they need to do to achieve improved quality of life. We gradually help them take up some interests, hobbies. The PRP obviously helps clients. It is just like students who submit their assignments to teachers. The PRP lists several steps that a client needs to take … I show them the PRP form every time when we meet. I then listen to clients who share their progress. It’s interesting. The clients follow the PRP and continue to work on it.”* (Case Worker: 190813_CS02_SMCM_108–109)

The service users had mixed feelings about how the SMCM case workers used the strengths assessment and PRP. One group of users felt that using the forms was helpful in structuring the case management process and increasing service users’ level of engagement in the recovery process.

*“Interviewer (I): Were the goals which were listed in the PRP form set by you and the worker together in the past year?*

*Service user (SU): Yes.*

*I: Do you remember what goals you set?*

*SU: The first goal is for me to pay attention to my diet and intake of calories. Second, I should take care of myself and my family. I should do some physical exercise, that is, the case worker suggested that I reserve some gym facilities to do some exercise at ABC (Centre’s name) Centre. I made an effort and did some exercise.*

*I: How did you feel when you saw the PRP form that the case worker photocopied for you?*

*SU: I felt good and I made changes. For example, I started doing physical exercise, something I hadn’t done for a long time. I went with my family to ABC (restaurant’s name) restaurant to have a meal together. I seldom talked to my family before, but now I talk to them more.”* (Service User: 190813_C070_LYW_SMCM_24–25)

*“I remembered the form which asked me about what I wanted in the future; that is, I should try to do something that I could do in the future. If I cannot do it, just leave it … it (the PRP form) is helpful. I think it helps me plan for the future … Yes, it encouraged me not to think of my past and not to lose hope for the future. It is helpful.”* (Service User: 190821_B012_LYK_SMCM_user_3.33)

However, case workers recalled that some service users found the use of the forms (e.g., the two-page, seven-domain strengths assessment) very stressful.

*“The effectiveness of the model relies on whether the client takes the initiative or whether they accept it. For example, I have 13 cases. Among them, some feel this model stresses them out. In some cases, when I took out the form, they said they felt pressurized and asked whether it was possible not to show it to them. You set the goals for them and they wonder why there are goals and whether they are being required to do something. It’s stressful.”* (Case Worker: 190721_CS21_SMCM_26)

## Discussion

Based on the comments of 24 Chinese service users and 14 case workers, the current study provides us with new knowledge about the perceived impacts and the experience of interventions (SMCM or control group) which was hitherto missing from the literature.

The qualitative data identified distinctive differences between the SMCM and usual care, in particular the improvements in the functional recovery of service users, such as looking for paid employment, widening their social circle, or making closer contact with their families, which suggests that some of the adverse effects of mental illness on the psychological, social and vocational domains were reduced. The current findings are consistent with the existing literature [16, 31–33] and indicate improvement in psychosocial outcomes, particularly better functioning and interpersonal relationships [34]. According to previous studies, the SMCM enables service users to be motivated in activities and improve their self-care ability, emotional self-management and interpersonal relationships [7, 35]. Such improvements can increase the chances of functional recovery and enhance positive thinking and perceived self-efficacy (e.g., feeling full of hope about recovery and accepting personal weaknesses or shortcomings; e.g., [36]). Furthermore, our results show that compared with usual care, the SMCM had added value: for example, improved communication skills; stronger self-appraisal on strengths, talents and skills; and the maintenance of proactive adherence and willingness to achieve the goals identified during the intervention, such as when going over the strengths assessment and PRP.

Our results show that service users in both groups (SMCM and usual care) stressed the importance of the working alliance between case workers and service users, suggesting that regardless of the type of intervention, a positive therapeutic working relationship is central to improved patient outcomes. In describing their experience of receiving the SMCM treatment, service users highlighted that case workers are like “co-walkers” who support them in their journey of recovery. Case workers guided service users to set and achieve their recovery goals and accompanied them through difficult times/circumstances in their personal lives. With social connectedness and supportive relationships being integral to well-being [37], the development of such a strong bond of trust between service users and case workers helps service users improve their well-being, a point which was also mentioned by the service users using usual care. In addition, the detailed accounts by service users underscore the attributes and skills of case workers that are essential to the therapeutic effects of the SMCM—being caring, helpful, approachable, and perseverant and maintaining regular contact with service users. On the basis of the results, future studies can further explore which aspect of the SMCM (e.g., low case worker to service user ratio) or which SMCM tools (e.g., strengths assessment) can promote the development of the working alliance, which could be useful for the field.

Having individual sessions of about 30 min every fortnight for the SMCM group seems to be a double-edged sword: On the one hand, it assists service users in their recovery; on the other hand, case workers reported that some dependence on case workers develops, and some service users considered it too demanding of their time. Therefore, not only the duration and frequency of individual sessions but also how the broader spirit or principles of the SMCM are put into practice require further investigation.

Participating service users and case workers highlighted some challenges to the successful implementation of the SMCM: the use of strengths assessment, the PRP, and group supervision. Service users had conflicting views on the use of the strengths assessment and PRP. While the majority considered that using the forms was helpful in achieving the set goals and could increase the level of partnership between service users and workers in the recovery process, some expressed that using the forms was demanding and very stressful for them. Therefore, it is recommended that case workers (1) improve their skills in using the strengths assessment and PRP through practice and gaining feedback through field mentoring with their supervisor and (2) observe service users’ level of readiness before using (and showing) the strengths assessment and PRP to identify and achieve goals.

Finally, one case worker expressed that their understanding of a service user may yield some subjective, skewed information that they may share during group supervision. The goal of group supervision, after all, is to share experiences, learn about what other colleagues are going through [25], and help generate creative, specific and useful strategies to assist service users to achieve their goals [29]. As many ideas/suggestions are given to case workers, they are encouraged to review and decide which idea is more suitable for implementing with service users.

### Implications for psychosocial interventions

First, given the positive outcomes of the SMCM, it is recommended that more community-based mental health centres consider adopting this approach to promote the recovery of individuals with mental illness and improve outcomes, because, as highlighted by case workers, it provides them with a useful professional tool to deliver services. This may be particularly helpful to case workers with less working experience; the SMCM tools would enhance their capability and self-efficacy to help service users in their recovery. To increase the scale of implementation of the SMCM, more training should be provided to case workers to help them master the relevant knowledge and skills. Second, the close relationship between service users and case workers is one of the key features of the SMCM found in the present study. According to previous studies, service users want case workers to be warm and calm, listen attentively, be responsive, show acceptance, be confident and understanding, and be well prepared for a session [38, 39]. It is recommended that case workers interact with service users to develop a good level of trust and to assist them in their recovery.

### Limitations

The present study has limitations. It was conducted in three community-based mental health centres in Hong Kong, so more community-based mental health centres should be included in future studies to enable comparison of the findings between different sites. Given that the samples were comprised of Chinese service users, the findings may be more culturally bounded and so may not be generalizable to other populations of individuals with mental health disorders from other cultures. Next, since only case workers and service users were interviewed in this study, the voices of service users’ families should be included in future studies to provide their perceptions of changes in service users since receiving the SMCM intervention. This would also help triangulate the findings reported by case workers and service users. Apart from retrospective interviews, future studies could also use longitudinal qualitative studies to capture service users’ and case workers’ experience of the SMCM over time.

## Conclusion

The results of the current study contribute important information about the experiences of service users and case workers, which are essential perspectives that inform strengths-based practices. This study helps us to better understand the implementation of the SMCM and informs us on how to improve service delivery, including the experiences of providing and receiving the intervention.

## Data Availability

Data are the transcripts of interviews containing identifiable information. Data are not publicly available due to concerns that the participants’ privacy may be compromised. Anonymized and de-identified data may be requested from the corresponding author.

## Abbreviations

SMCM: Strengths model of case management
RCT: Randomized controlled trial
ICCMW: Integrated community centre for mental wellness
PRP: Personal recovery plan
